# An Experiment Assessing Punitive versus Wellness Framing of a Tobacco-Free Campus Policy on Students’ Perceived Level of University Support

**DOI:** 10.3390/ijerph14080938

**Published:** 2017-08-20

**Authors:** Joseph G. L. Lee, Christopher J. Purcell, Beth H. Chaney

**Affiliations:** 1 Department of Health Education and Promotion, College of Health and Human Performance, East Carolina University, Mail Stop 529, 1000 E. 5th St., Greenville, NC 27858, USA; chaneye@ecu.edu; 2 Department of Leadership, Policy, and Organizations, Peabody School of Education, Vanderbilt University, PMB #414, 230 Appleton Place, Nashville, TN 37203, USA; chris.purcell@vanderbilt.edu

**Keywords:** universities, health policy, communication, smoke-free, organizational support

## Abstract

The objective of this study was to examine how different ways of describing a hypothetical tobacco-free campus policy would impact college students’ perceived level of support from the college. In the spring of 2016, we randomized 1885 undergraduate students in a required course to three message conditions in an online survey: control (no message), wellness (emphasizing promoting health and quitting support), and punitive (emphasizing consequences for violating the policy). The dependent variable was perceived organizational support. We selected items previously shown to be relevant for college students (alpha = 0.92 in our data). Given significant non-normality, we used non-parametric Kruskal-Wallis tests with pairwise comparisons to examine differences in perceived organizational support across the three conditions. We examined results by smoking status and if the participant correctly reported the message they received. We found no significant difference in perceived organizational support among students exposed to different tobacco-free campus policy announcements (*p* = 0.75). We also found no significant difference among smokers (*p* = 0.66). However, among smokers who correctly reported the message they received, we found significantly lower perceived university support (*p* = 0.01). Messages about tobacco-free campus policies should focus on the role of policy in supporting a healthy environment instead of punitive enforcement. Campus administrators should use caution when using message frames focusing on consequences of violating newly adopted policies.

## 1. Introduction

The national movement for tobacco-free college and university policies was prompted by a period of sharp uptake in tobacco use among college students in the 1990s [1]. This movement remains important given the use of alternative tobacco products, such as hookah and electronic cigarettes [2,3]. Tobacco-free policies including all buildings and grounds are recommended by the American College Health Association [4]. Such policies have four benefits: (1) they protect students from involuntary smoking or second-hand smoke [5], (2) they likely reduce the prevalence of smoking [6], (3) they likely reduce maintenance costs and risk of fires on campus, and (4) they create an environment that can help college students develop into healthy adults [7,8]. As of 3 July 2017, over 1600 U.S. college campuses were tobacco-free [9].

While researchers have documented ways to increase compliance with tobacco-free campus policies [10,11,12], there is less information about best practices in communicating tobacco-free policies to the campus community. How policies are framed—that is, how they are linked and organized with other social and cultural themes—can change how they are viewed and supported in the political process [13]. Framing can also change compliance with policies [14,15]. For example, adoption of a tobacco-free campus policy with a frame of wellness promotion could enhance the reputation of a campus as a place that cares about student well-being. Conversely, adoption of a tobacco-free campus policy with a frame of punitive enforcement could make tobacco users feel ostracized and “picked on”. Different approaches to message framing could help or hinder tobacco-free initiative outcomes including compliance, cessation, and support for the policy. Framing used in policy implementation could also advance other goals of university administrators. These could include improving how supported the campus community feels by the institution, building synergy with an institutional mission to promote health, and improving indicators of how the campus community feels about the college or university.

The purpose of this study is twofold: (1) to experimentally assess the role of tobacco-free campus message framing on students’ perceived university support and (2) to identify whether responses to framing are different among smokers.

## 2. Materials and Methods 

### 2.1. Methods

East Carolina University (ECU) is a large regional research university located in the U.S. state of North Carolina, where there is a substantial amount of tobacco farming. Currently, campus policy does not allow smoking inside or within 25 feet of university-controlled buildings. Undergraduate students enrolled in a required course were recruited with in-class announcements and via e-mails through the course management system. Recruitment was conducted during the spring 2016 semester. Enrolled students were provided a link to participate in an online survey administered using Qualtrics© (Provo, UT, USA). Student participants were asked to provide electronic informed consent prior to taking the survey. The final page of the survey could be printed and turned in for extra credit. Other extra credit opportunities were available to students, and protections were in place to prevent coercion or undue inducement to participate. The East Carolina University and Medical Center Institutional Review Board exempted the study from further review (#16-000443). Of the 2298 enrolled students, 1885 completed the full survey and responded to the policy support questions (response rate = 82%). 

### 2.2. Measures

#### 2.2.1. Student Characteristics

Measures used for student characteristics included cigarette use in the past month (“In the past 30 days, on how many days did you smoke a cigarette (even a puff)?”), whether they have smoked 100 cigarettes in their lifetime, if they currently smoked (every day, some days, not at all), gender (“Do you consider yourself?” (male, female, other)), and class year.

#### 2.2.2. Experimental Stimuli

We used advanced block randomization in Qualtrics. After the survey consent and demographic questions, participants received the following section heading: “PERCEPTIONS: The following section has questions about policies, communication, and smoking”. After this, participants were randomized to one of the three following messages (Appendix A): (1) a control message simply instructing them to continue to the next page, (2) a hypothetical new policy announcement message highlighting the university’s support for quitting and desire to promote wellness, or (3) a hypothetical new policy announcement message highlighting consequences for those not complying with the new policy. Messages 2 and 3 were based off of an actual formal notice e-mailed to all students at the University of North Carolina at Chapel Hill [16]. 

#### 2.2.3. Manipulation Check

Online surveys can be completed quickly and may be done in high-distraction environments, such as in a café or while watching television. Manipulation checks are a method to verify whether the participants noticed the experimental stimulus. Thus, we included a manipulation check for message conditions 2 and 3. We did not include a manipulation check for the control condition, as it was the absence of a message. Manipulation checks were conducted to ensure that the most salient parts of the message condition were received by the participant. The manipulation check read, “When you read about a tobacco-free policy earlier, what was the most important part of the message?” Responses were presented in random order and read as follows: (a) Help was available for those who want to quit tobacco, (b) There are consequences for people who smoke on campus, (c) Tobacco use is bad for health, and (d) Don’t know/Don’t remember. Passing the manipulation check for the wellness and punitive messages was calculated as selecting either response (a) or (b), respectively. Those not passing the manipulation check were omitted from the analyses designed to include only those who could be confirmed to have noticed the experimental stimuli. 

#### 2.2.4. Dependent Variable

For the dependent variable, we used an organizational support scale [17] that has been robustly tested for validity and is commonly used in organizational psychology [18,19]. The scale was originally developed to assess employees’ “beliefs concerning the extent to which the organization values their contributions and cares about their well-being” [17]. The scale is strongly related to employee satisfaction, commitment, and intention to stay in or leave the organization [19]. Previous research modified and tested the scale for use in college student populations to assess perceived university support of students, finding a significant relationship with school-life satisfaction [20], loyalty or commitment to the university [21], and intention to complete their degree [22]. 

The seven items were presented in random order with seven-response options (strongly disagree to strongly agree). The items were: (a) ECU really cares about my well-being, (b) Help is available from ECU when I have a problem, (c) ECU tries to make my school life as interesting as possible, (d) ECU is willing to extend itself to help me perform to the best of my ability, (e) ECU cares about my general satisfaction at school, (f) ECU shows very little concern for me (reverse coded), and (g) ECU takes pride in my accomplishments at school. We summed and divided by seven for our measure of perceived organizational support. Internal consistency of the scale was strong in our data (α = 0.92).

### 2.3. Data Analysis

Data analysis was conducted in SPSS 24 (IBM Inc., Chicago, IL, USA) in the fall of 2016. Descriptive statistics were computed to summarize participant characteristics, and Chi-square tests assessed if characteristics were distributed across the three conditions. Because of non-normality in the dependent variable, non-parametric Kruskal-Wallis tests were used in lieu of ANOVA. Pairwise comparisons used a Bonferroni correction. Differences were examined between: (1) the three conditions overall; (2) the three conditions among participants who reported smoking in the past month; (3) the three conditions among participants who correctly answered the manipulation check or were in the control condition; and (4) the three conditions among participants who reported smoking in the past month and correctly answered the manipulation check or who reported smoking in the past month and were in the control condition. We calculated means and 95% confidence intervals using SPSS Compare Means with bootstrapping. Missing data were rare; we used pairwise deletion. Alpha was set a priori at 0.05, and two-tailed statistical tests were conducted.

## 3. Results

The participant characteristics of the 1885 completed surveys are presented in Table 1 by experimental condition. There were no differences between conditions in student characteristics; however, the manipulation check was more likely to be passed by those in the wellness condition. Perceived organizational support was high (M = 5.46, SD= 1.23). Differences in the dependent variable by condition are presented in Table 2. There were no significant differences between the three conditions in perceived organizational support (*x^2^* = 0.58, df = 2, n = 1885, *p* = 0.75). There were no significant differences between the three conditions in perceived organizational support among participants who had reported smoking in the last month (*x^2^* = 0.85, df = 2, n = 329, *p* = 0.66). There were no significant differences between the conditions among those who passed the manipulation check or were in the control group (*x^2^* = 1.41, df = 2, n = 868, *p* = 0.49). However, among participants who smoked in the past month and passed the manipulation check (n = 45) or smoked and were in the control condition (n = 118), there were significantly differences (*x^2^* = 9.52, df = 2, n = 163, *p* = 0.01). For these participants, pairwise comparisons with Bonferroni correction showed significant differences between the punitive vs. supportive message (*p* = 0.01), and between the punitive and control condition (*p* = 0.02), but not between the supportive and control condition (*p* = 0.97). The same pattern of direction and significance was observed using a more stringent definition of smoking (100 cigarettes in lifetime as well as every day or some days smoking now, data not shown). 

## 4. Discussion

### 4.1. Discussion

We found that messages about a tobacco-free campus policy focusing on wellness had no adverse impact on an important indicator of student success, perceived organizational support. Yet, messages focusing on punitive enforcement of a tobacco-free campus policy had a negative impact on perceived organizational support among students who had smoked in the past month and passed a manipulation check. That is, the students who would be most impacted by a tobacco-free policy showed lower levels of perceived organizational support when shown punitively-framed messages about a hypothetical tobacco-free policy. 

These participants represent a portion of the campus community that is important to support in efforts to address tobacco use. Our results indicate that campus administrators and health/wellness professionals should consider framing tobacco-free campus policies as part of an effort to promote health and wellness. This finding is supported by studies of how smoke-free policies are communicated; one 1981 study showed better compliance with positively rather than negatively worded no-smoking signs in a hospital waiting room [15]. A more recent study showed employees’ organizational attraction was helped by a workplace policy that emphasized support for quitting compared to one without any support mentioned [23]. 

As argued by Hahn and colleagues, implementation of a tobacco-free campus policy requires three T’s: Telling the campus about the policy, providing Treatment to support quitting, and Training the campus community on how to promote compliance [24]. This study adds to the literature by providing evidence that communication efforts should focus on the reason for the policy (promoting health and wellness). Nonetheless, clear communication about the expectations of compliance with the policy is still warranted. Indeed, the perceived organizational support scale maps conceptually onto institutional best practices in student retention [25]. One influential theory of student persistence in residential colleges and universities points to 13 forces that demonstrate a commitment of the institution to the welfare of its students, including organizational behavior [26]. The authors state, “students observe actions involving communication of rules and requirements, fairness in the administraton of rules and requirements, and the provision of opportunities to participate in making decisions regarding matters of importance to students” (p. 103). The authors assert, “If students feel well informed about rules and requirements pertinent to student life, they perceive that their institution places a high value on students” (p. 103). 

Student conduct and campus wellness officials should coordinate efforts to clearly state policies and the resources available to the campus community. Students expect campus policies to be enforced [27], but framing messages to focus on consequences may prompt deleterious effects among the very student population most in need of university support. Future research should examine the framing of efforts to promote compliance after the policy is implemented. 

Our non-significant findings may stem from high levels of student support for tobacco-free campus policies. Policy support for tobacco-free campus policies is high [28]: Berg and colleagues reported 63.3% of undergraduate students at a large Midwestern public university had positive views of a smoke-free campus policy’s impact on student quality of life [29]. At a public university in the Pacific Northwest, 72% of students reported supporting a smoke-free campus [30]. At eight California public universities, regardless of current policies, over two thirds of students thought that regulation of smoking in outdoor places was a “good thing” [31]. In the tobacco-growing region of Eastern Tennessee, researchers found lower levels of support among tobacco users, but a substantial portion of tobacco-using participants were supportive, approximately 20% [32]. 

### 4.2. Limitations

This research has a number of limitations. First, this research may not be generalizable to other universities and student populations. Second, our experimental survey methods do not replicate a real-world roll-out of messaging about policy change. Third, the relatively small number of current smokers in our sample is cause for concern about the reproducibility of our study. Fourth, we did not have data on electronic nicotine delivery system use or use of other tobacco products, which should be addressed in future research. Fifth, the relatively small proportion of participants passing our manipulation check suggests that future research should use stronger stimuli. Additionally, the manipulation check was passed significantly more frequently among the wellness condition than the punitive condition. This may be due to better congruence between the wellness frame and wellness manipulation check response option. Sixth, the survey was administered as part of a required course on health; taking the survey as part of a health class may prompt a greater focus on health for respondents.

## 5. Conclusions

Higher education administration research approaches tobacco-free campus policies as a way of promoting college students’ development of healthy lifestyles from a social-ecological framework [7,33]. Our findings suggest that campus health officials and administrators implementing tobacco-free policies should leverage this focus on a health-promoting environment [34]. Tobacco-free campus policies framed to be about wellness do no harm to perceived organizational support, a key indicator of interest to university administrators that is tied to student retention. However, when framed to focus on punitive consequences for policy violators, messages about the adoption of a tobacco-free campus policy can prompt reductions in perceived organizational support among smokers. Campus administrators should link tobacco-free campus policies to institutional efforts to promote wellness.

## Figures and Tables

**Table 1:** Study participants, college students (n = 1885), spring 2016.

	Control Condition (n = 632)	Punitive Condition (n = 625)	Wellness Condition (n = 628)	Total (%) *	*p* for Difference across Conditions
Gender					0.53
Female	402 (64%)	393 (63%)	382 (61%)	1177 (62%)	
Male	229 (36%)	230 (37%)	245 (39%)	704 (37%)	
Other	0	1 (0.2%)	0	1 (0.1%)	
School Year					0.26
First year	479 (76%)	479 (77%)	474 (76%)	1432 (76%)	
Sophomore	92 (15%)	89 (14%)	91 (15%)	272 (14%)	
Junior	52 (8%)	42 (7%)	44 (7%)	138 (7%)	
Senior	5 (1%)	10 (2%)	17 (3%)	32 (2%)	
Other	2 (0.3%)	5 (0.8%)	2 (0.3%)	9 (1%)	
Any smoking, past month	118 (19%)	94 (15%)	117 (19%)	329 (18%)	0.14
Current smoker **	58 (9%)	43 (7%)	53 (8%)	154 (8%)	0.91
Passed manipulation check (message conditions only)	-	92 (15%)	144 (23%)	236 (19%)	0.001

Note: * Frequencies do not sum up to 1885 due to sporadic missingness and percentages may not add to 100 due to rounding, ** 100 cigarettes in lifetime and now smoke every day or some days.

**Table 2:** Perceived organizational support by condition, college students, spring 2016.

Analysis	Control: No Message			Punitive Message			Wellness Message		
	N	Mean	(95% CI)	N	Mean	95% CI	N	Mean	95% CI
All Participants	632	5.49	5.40–5.59	625	5.45	5.36–5.55	628	5.43	5.33–5.52
Smokers	118	5.26	5.03–5.50	94	5.12	4.84–5.39	117	5.21	4.99–5.43
Passed manipulation check	-	-	-	92	5.49	5.20-5.76	144	5.62	5.40-5.81
Smokers who passed manipulation check or in control condition	118	5.26 ^a^	5.03–5.49	16	4.20 ^b^	3.46–4.95	29	5.47 ^a^	4.95–5.98

*Note*: Presence of superscript letter indicates statistically significant Kruskal-Wallis test; ^a,b^ significant differences between columns using pairwise comparisons with Bonferroni correction are indicated by different letters. Means and confidence intervals are calculated with bootstrapping.

## Appendix A

Appendix A contains the messages used in this study.

**Appendix A:** Control, punitive, and supportive messages used in experiment.

Please continue to the next question.
**Please read the following hypothetical message from campus administrators:** FORMAL NOTICE To: All Students, Faculty, and Staff: After consultation with faculty, students, and staff, the University has adopted a new policy regarding tobacco use on campus. While we have banned smoking inside University buildings and facilities for years, we will expand the policy to all University facilities, both on and off campus, and we will not have any designated smoking areas. **The practical effect of this policy is that the campus will be tobacco-free.** The University policy applies to all University faculty, staff, students, visitors, and patients. We have begun posting signs throughout campus to ensure that everyone is aware of the tobacco-free policy. People who violate the tobacco ban will be asked by the University’s public safety officers to stop smoking. **Anyone who refuses to comply or has been warned previously is at risk of referral to student conduct or human resources**.
**Please read the following hypothetical message from campus administrators:** FORMAL NOTICE To: All Students, Faculty, and Staff: The University is pleased to announce a new policy following consultation with the faculty, students, and staff as part of our efforts to promote the health and well-being of all members of the campus community. While we have banned smoking inside University buildings and facilities for years, we will expand the policy to all University facilities, both on and off campus, and we will not have any designated smoking areas. **The practical effect of this policy is that the campus will be tobacco-free.** The intent of the policy is to promote the health and well-being of people on our campus. We have begun posting signs throughout campus to ensure that everyone is aware of the tobacco-free policy. As this policy may be stressful for some of our faculty, staff, and students who choose to use tobacco, we will provide resources to those who would like to quit smoking. Help quitting is available from both student health, employee health, and by phone (1-800-QUITNOW). **We have created a small fund to help provide nicotine replacement therapy for students whose insurance does not cover it.**
